# miR-340 suppresses glioblastoma multiforme

**DOI:** 10.18632/oncotarget.3288

**Published:** 2015-03-16

**Authors:** Daquan Huang, Shuwei Qiu, Ruiguang Ge, Lei He, Mei Li, Yi Li, Ying Peng

**Affiliations:** ^1^ Department of Neurology, Sun Yat-sen Memorial Hospital, Sun Yat-sen University, Guangzhou, China; ^2^ Guangdong Provincial Key Laboratory of Malignant Tumor Epigenetics and Gene Regulation, Sun Yat-Sen Memorial Hospital, Sun Yat-Sen University, Guangzhou, China; ^3^ Key Laboratory of Gene Engineering of the Ministry of Education and State Key Laboratory of Biocontrol, College of Life Sciences, Sun Yat-Sen University, Guangzhou, China

**Keywords:** glioblastoma multiforme, miR-340, ROCK1, biomarker

## Abstract

Deregulation of microRNAs (miRs) contributes to tumorigenesis. Down-regulation of miR-340 is observed in multiple types of cancers. However, the biological function of miR-340 in glioblastoma multiforme (GBM) remains largely unknown. In the present study, we demonstrated that expression of miR-340 was downregulated in both glioma cell lines and tissues. Survival of GBM patients with high levels of miR-340 was significantly extended in comparison to patients expressing low miR-340 levels. Biological functional experiments showed that the restoration of miR-340 dramatically inhibited glioma cell proliferation, induced cell-cycle arrest and apoptosis, suppressed cell motility and promoted autophagy and terminal differentiation. Mechanistic studies disclosed that, miR-340 over-expression suppressed several oncogenes including p-AKT, EZH2, EGFR, BMI1 and XIAP. Furthermore, ROCK1 was validated as a direct functional target miR-340 and silencing of ROCK1 phenocopied the anti-tumor effect of mR-340. Our findings indicate an important role of miR-340 as a glioma killer, and suggest a potential prognosis biomarker and therapeutic target for GBM.

## INTRODUCTION

Glioblastoma multiforme (GBM) is the most common and deadly primary brain tumor in adults, characterized with highly invasion, rapid cell growth and resistance against apoptosis. Despite the aggressive treatment with combination of surgery, radiotherapy and chemotherapy, the overall prognosis remains poor with a median of survival of 10–14 months [1–3]. Therefore, it is urgent to figure out the precise molecular mechanism of its pathogenesis and explore novel therapeutic strategies to treat this devastating disease.

The uncontrollable growth and invasive behavior could be responsible for the deadly nature of malignant glioma. However, Reprograming the cells’ fate to terminally differentiation is one potential approach to dampen tumor growth and invasion. During the differentiation process of glioma cells, the mature glial marker GFAP or the neural-specific transcriptional factors including DLX2, Brn3a and NeuroD6 [4], are up-regulated along with the specific morphological changes and weak proliferative and invasive capacity. Previous studies report the glioma cells are induced toward terminal differentiation by cholera toxin [5], all-trans retinoic acid [6], induction of autophagy [7], activation of BMP signaling [8]. Induction of differentiation consequently domesticates the malignancy of cancers back to normal way. Hence, finding the potent differentiation agents remains a real challenge for malignant glioma [9, 10].

Growing evidence confirmed that miRNAs play essential roles in cancer etiology. MiRNAs are involved in diverse biologic processes including cell growth, migration and invasion, apoptosis and autophagy as well as differentiation [11–15]. Multiple miRNAs have been reported to be dysregulated in GBM and function as tumor suppressors [16]. Our group has previously reported that the tumor suppressor miR-138 inhibits glioma growth through targeting EZH2 mediated signaling pathway associated with cell proliferation and cell cycle [17]. Deregulation of miR-340 is observed in different human tumors [18–22]. It has been shown that up-regulation of miR-340 inhibits the migration and invasion of breast cancer and osteosarcoma [18, 20]and suppresses cell proliferation of non-small cell lung cancer and colorectal cancer [19, 21], indicating that miR-340 exerts as one tumor suppressor in tumorigenesis. To our knowledge, however, whether miR-340 is involved in glioma cell differentiation is not reported. Besides, miR-340 is reduced in glioma according to two microarray-based analysis [23, 24], whereas biological function of miR-340 in glioma tumorigenesis remains largely unknown. Therefore, functions of miR-340 on gliomagenesis and underlying molecular mechanisms were investigated in our studies.

Here, we reported that miR-340 was correlated with survival of GBM patients. MiR-340 inhibited cell growth and motility and induced the terminal differentiation through modulation of hallmarks of glioma cells. Furthermore, ROCK1 was validated as a direct functional target miR-340 in glioma and silencing of ROCK1 phenocopied the anti-tumor effect of mR-340.

## RESULTS

### MiR-340 expression is reduced in glioma cells and tissues and correlates with patient survival

**Figure 1:**
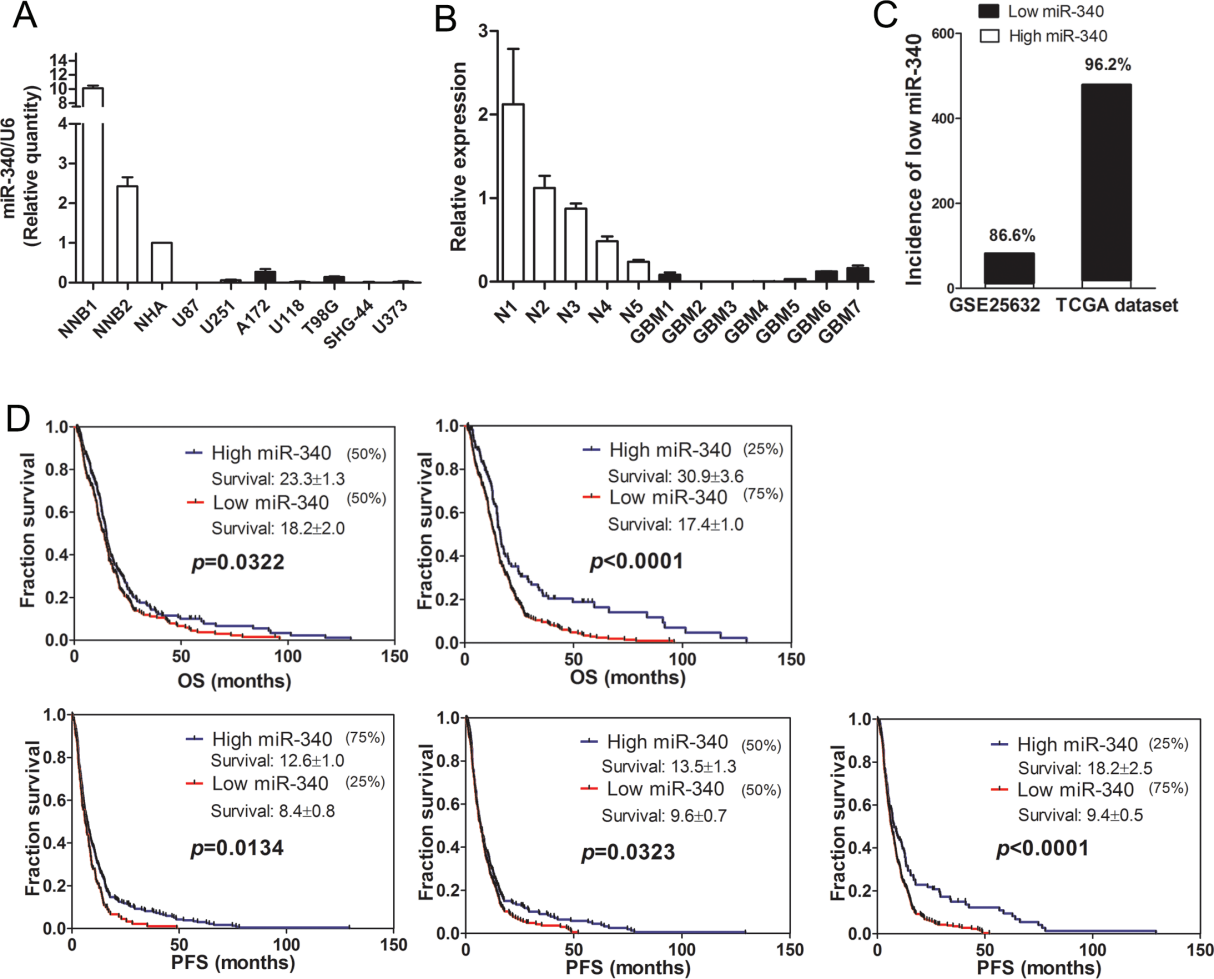
Expression of miR-340 is downregulated in glioma and related with survival of glioma patients **A.** The expression of miR-340 in seven glioma cell lines was measured by qRT-PCR, two non-neolpastic brains (NNBs) and NHA were used as controls. **B.** The expression of miR-340 in five normal brain tissues and seven GBM tissues. **C.** The incidence of low levels of miR-340 in GBM was evaluated using two microarrays, GEO dataset GSE25632 and TCGA dataset. **D.** Overall survival (OS) and progression free survival (PFS) curves of Kaplan-Meier analysis. 482 expression values of miR-340 were sorted by ascending order and OS were analyzed at different stratifications of quartiles of miR-340 expression levels. High level of miR-340 was related to long OS among the TCGA dataset at the indicated cut-off. The Log-rank test *p* value for the difference between two survival curves for the miR-340-high and – low expression GBM patients was indicated.

First, miR-340 levels were measured in normal human astrocytes (NHA), normal brain tissues and glioma cell lines. The result showed that level of miR-340 in glioblastoma cells was significantly lower than that in NHA and normal brain tissues (Figure 1A). Second, miR-340 level were also tested between GBM tissue and normal brains, we found that the expression of miR-340 in GBM tissues was reduced compared with the normal brains (Figure 1B). Furthermore, the incidence of low levels of miR-340 in GBM was evaluated using two microarrays, GEO dataset GSE25632 and TCGA dataset. Using the mean expression level of miR-340 in normal brain tissues as the cut-off, the incidence of low miR-340 was 86.6% (71/82) in GSE25632 data, while one higher incidence of 96.3% (464/482) was displayed in TCGA dataset (Figure 1C), implying that the frequent reduction of miR-340 plays important roles in GBM progression.

Next, in virtue of the TCGA data, association between miR-340 expression and post-diagnosis survival were investigated in GBM patients using the Kaplan-Meier survival analysis. At diverse quartile stratifications, low expression levels of miR-340 were significantly correlated with short overall survival (OS) and progression free survival (PFS) in comparison to high miR-340 levels; In particular, at the 75% stratification, low expression levels of miR-340 apparently shorten survival of both OS and PFS compared to high levels of miR-340 (median OS: 30.9 vs 17.4 months; median PFS: 18.2 vs 9.4 months; *p* < 0.0001; Figure 1D). Moreover, low miR-340 was also related with unfavorable clinical outcome of patients with survival longer than 2 years compared with high miR-340 (Supplementary Figure S1). These survival data suggested a valuable prognosis biomarker of miR-340 for GBM patients and miR-340 might participate in gliomagenesis.

### Overexpression of miR-340 leads to reduction of glioma cell growth

**Figure 2:**
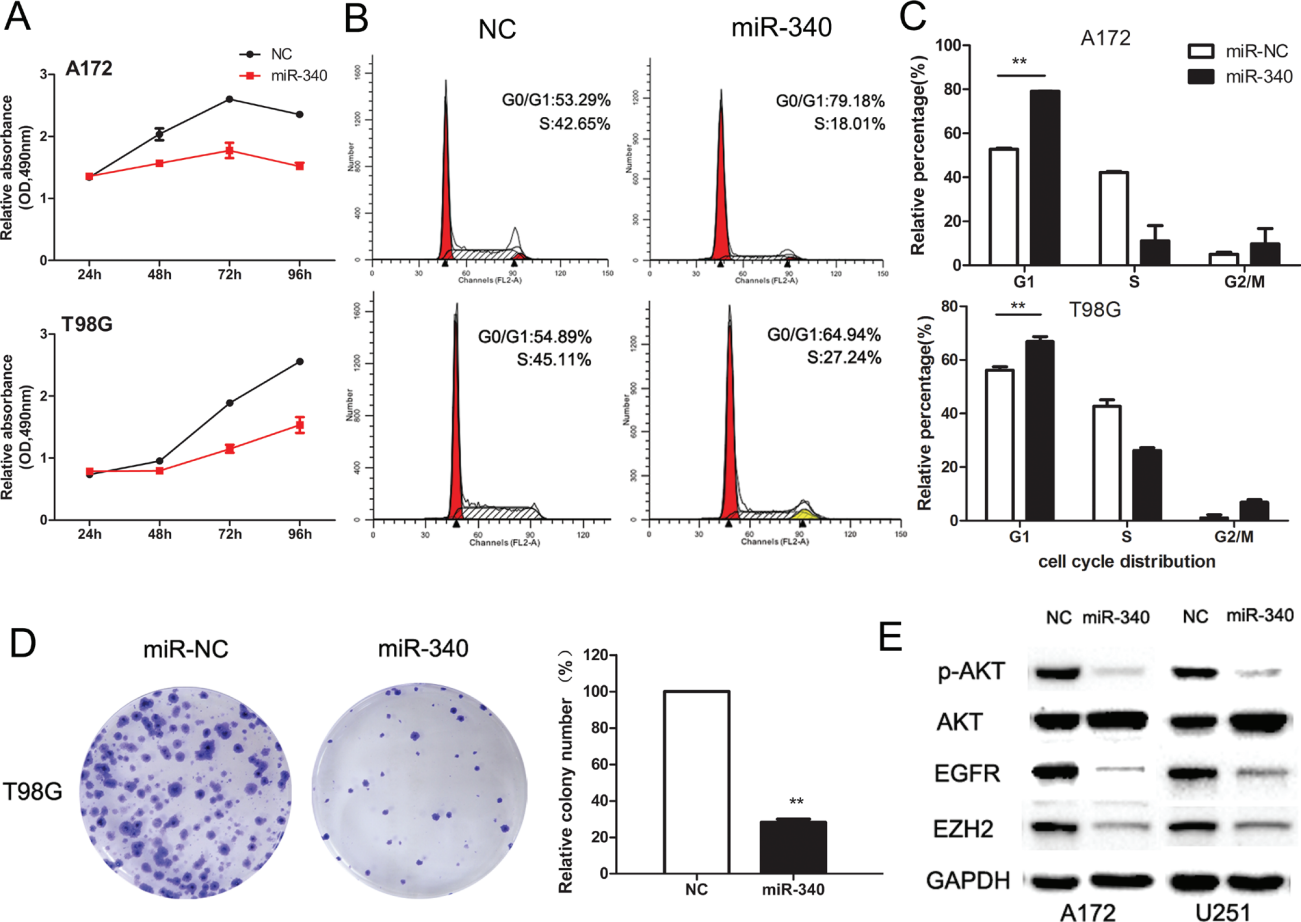
Enforced expression of miR-340 induces growth inhibition in glioma cells **A.** Effect of miR-340 on cell viability was measured by MTS assay after miRNA transfection in A172 and T98G cells. **B.** Representative histograms for cell-cycle of A172 and T98G cells transfected with miRNAs for 72 h. **C.** Statistic analysis of cell cycle distributions from three repeating experiments. **p < 0.01. **D.** Representative pictures of colony formation asssy of T98G transfected miR-340 or NC. Colonies were evaluated and values were reported as the ratio between the indicated transfected cells. **p < 0.01 compared with control. **E.** Western blots of p-AKT (ser473), AKT, EGFR, EZH2 levels in A172 and U251 cells transfected with NC or miR-340 for 72 h.

To explore the biological function of miR-340 on glioma cells growth, glioma cells were tranfected with miR-340 mimics and cell viability were measured by MTS assay. The results showed that miR-340 inhibited cell growth in T98G cells by 41% and in A172 cells by 36% (Figure 2A). Moreover, with the colony formation assay reflecting the cell proliferation, T98G cells transfected with miR-340 displayed much fewer and smaller colonies compared with Control cells (Figure 2D). Further mechanism analysis showed that overexpression of miR-340 caused cell-cycle arrest at G1/S phase (Figure 2B and 2C), resulting in cell growth retard.

Given the significant inhibition of cell growth by miR-340 on glioma cells, involvements of several genes related with cell proliferation and cell-cycle were investigated. As we have previously found that EZH2 plays a key role in regulation of glioma cell-cycle machinery [17], interestingly, we found that miR-340 significantly down-regulated EZH2 protein level as well. Moreover, expression of phosphorylated-AKT (p-AKT) and EGFR, which are well-known oncogenes playing essential roles in control of glioma cell proliferation, were obviously decreased by miR-340 (Figure 2E). In addition, miR-340 also reduced the expression level of CCND1 (Supplementary Figure S2), another key regulator of cell-cycle machinery. Collectively, these functional and mechanistic studies implicated that miR-340 was extensively involved in cell growth.

### MiR-340 inhibits the motility of glioma cells

**Figure 3:**
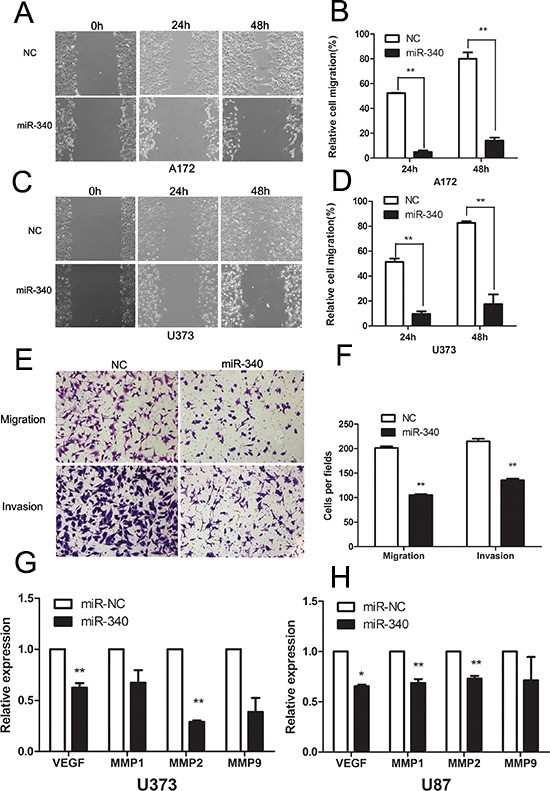
Overexpression of miR-340 inhibits glioma cells migration and invasion **A and C.** Representative images of wound healing assay were taken at different time points. (Original magnification: × 100). **B and D.** Quantification of cell motility was conducted by measuring the wound width. The amount of motility was displayed as a percent of migration cells at the time point 0. **p < 0.01. **E.** The migration and invasion abilities of U87 cells transfected with miR-340 or NC in the transwell assay. Representative migratory and invasive images are shown. (Original magnification: × 100). **F.** Quantitative analysis of the invasion and migratory cells from three independent experiments. **p < 0.01. **G and H.** Introduction of miR-340 reduced the mRNA levels of VEGF, MMP1, MMP2 and MMP9 in U87 and U373.

Next, to test the influence of miR-340 on the motility ability of glioma cells, a wound healing assay was employed to examine the effect of miR-340 on cell migration. As illustrated in Figure 3A and 3C, miR-340-transfected cells displayed apparently slower migration in relevant to control cells. Quantification of wound closure showed that after 24 and 48 hours, miR-340-transfected glioma cells closed 4.9% and 13.9% of the wound for A172 cells, and 9.7% and 17.5% for U373 cells, respectively, while miR-control glioma cells closed 52.3% and 80% of the wound for A172 cells, and 51.3% and 82.6% for U373 cells, respectively (Figure 3B and 3D). Furthermore, Transwell migration assays revealed that the restoration of miR-340 expression significantly restrained both glioma cell migration and invasion (Figure 3E and 3F). Furthermore, underling molecules related with motility were investigated and down-regulation of VEGF, MMP1, MMP2 and MMP9 were observed after restoration of miR-340 in U87 and U373 cells (Figure 3G and 3H). Taken together, these results indicate that miR-340 significantly inhibits migration and invasion of glioma cells.

### Apoptosis and autophagy are induced in response to miR-340 restoration

**Figure 4:**
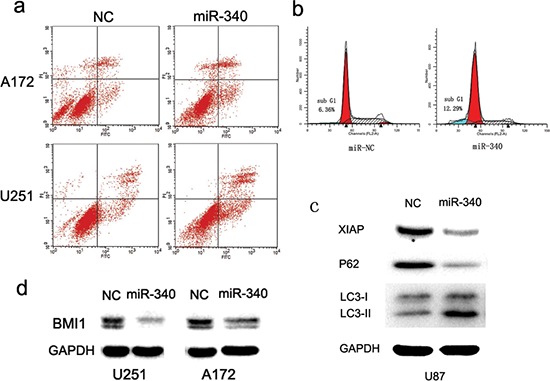
MiR-340 promotes glioma apoptosis and autophagy **A.** A172 and U251 were double stained with Annexin V-FITC/PI at 72 h post-transfection. Right lower quadrant shows early apoptosis cells, whereas right upper shows necrotic and terminal of apoptosis cells. **B.** MiR-340 induced accumulation of glioma cells in sub-G1. A172 cells were analyzed by PI staining. Fractions of apoptosis cells (sub-G1) are indicated by blue staining. **C.** Overexpression of miR-340 leads to autophagy, as determined by immunoblots with indicated antibodies. **D.** The expression level of Bmi-1 was decreased by transfection with miR-340 in U251 and A172, indicated by western blot.

Programmed cell death may also contribute to reduction of glioblastoma cell growth by over-expression of miR-340. To test this hypothesis, we investigated the effect of miR-340 on early apoptosis of glioma cells using the Annexin-V/propidium iodide(PI) assay and FCS, and found that the percentage of early apoptosis cells were greatly increased in both A172 and U251 cells transfected with miR-340 (Figure 4A). Besides, DNA degradation is a hallmark of apoptosis procedure, and thus cellular DNA content in A172 cells transfected with miR-340 mimics were detected using PI staining and the subsequent FCS results showed that A172 cells treated with miR-340 displayed strongly increased levels of sub-G1 population representing mostly apoptosis cells, compared to the control group (Figure 4B). These results indicate a potent apoptosis response of glioma cells to miR-340 restoration.

Induction of autophagy is one another mechanism by which tumor suppressors induce tumor growth inhibition. We found that the restoration of miR-340 expression resulted in significantly accumulation of LC3-II and down-regulation of p62 protein in U87 cells, indicators of autophagy (Figure 4C). In addition, XIAP has been found to an important anti-autophagy factor in tumor cells [25]. BMI1 is essential for glioma proliferation and self-renewal [26, 27]. We found that up-regulation of miR-340 markedly reduced XIAP and BMI1 protein expression (Figure 4C and 4D). Altogether, these results reveal an involvement of miR-340 in the regulation of apoptosis and autophagy of glioma cells.

### MiR-340 induces glioma cells towards terminal differentiation

**Figure 5:**
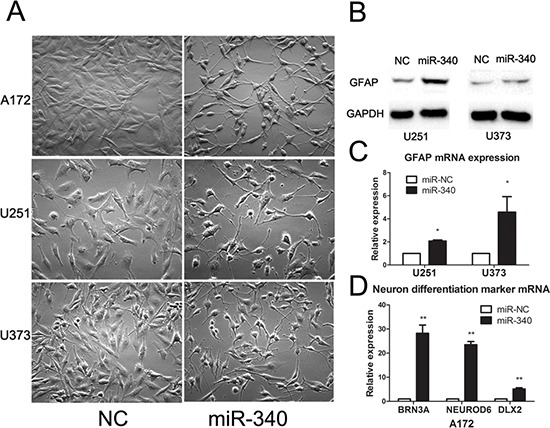
MiR-340 induces glioma morphology changes and upregulates the mature differentiation markers **A.** miR-340 induces morphological transformation of glioma cells. Indicated glioma cell lines were transfected with NC or miR-340 for 48 h (Original magnification: × 200). **B and C.** Effect of miR-340 on GFAP expression in glioma cells. **D.** miR-340 promotes the expression of neural-specific transcriptional factors DLX2, Brn3a and NeuroD6 in A172.

Microscopic observation of a panel of glioma cell lines transfected with miR-340 mimics displayed apparent changes in their morphology. Compared to the polygonal morphology of control cells, glioma cells transfected with miR-340 possessed smaller round cell bodies and much longer, fine and tapering processes, similar to mature neurocyte (Figure 5A). These morphological observations strongly indicates that miR-340 is capable of promoting terminal differentiation of glioma cells towards the maturation process of neurocyte.

Thus, the results above inspired us to examine the expression of glial fibrillary acid protein (GFAP), a reliable molecular marker of mature astrocytes. Western blotting showed that GFAP expression was significantly up-regulated in miR-340 treated cells relevant to control cells (Figure 5B). Transcription level of mRNA of GFAP was tested by qRT-PCR and the results revealed that expression of GFAP was also increased at the mRNA level (Figure 5C). As A172 cell line rarely express GFAP protein, examined by western blot (data not shown), we next evaluated expression of three well-established markers of neuron differentiation, DLX2, Brn3a and NeuroD6, which are neural –specific transcriptional factors. Real time qRT-PCR analysis showed that miR-340 restoration remarkably increased all these three factors (Figure 5D). Therefore, these data strongly suggested that miR-340 was able to induce differentiation of glioma cells, leading to less malignancy grade of GBM.

### ROCK1 is a direct target gene of miR-340

To delineate the mechanism of miR-340 for glioma inhibition, we searched for the predicted target genes of miR-340 from Targetscan database. ROCK1, the serine/threonine kinase that is involved in many aspects of cell behavior, was one of the potential candidates. We focused on ROCK1 since there are increasing evidence that ROCK1 plays an important role in growth, survival and invasion of tumor cells [28–30] and that it is also involved in the regulation of autophagy, apoptosis and differentiation [31–34]. In addition, ROCK1 is found to be highly expressed in glioma and correlated with the degree of malignancy of astrocytic tumors [35].

**Figure 6:**
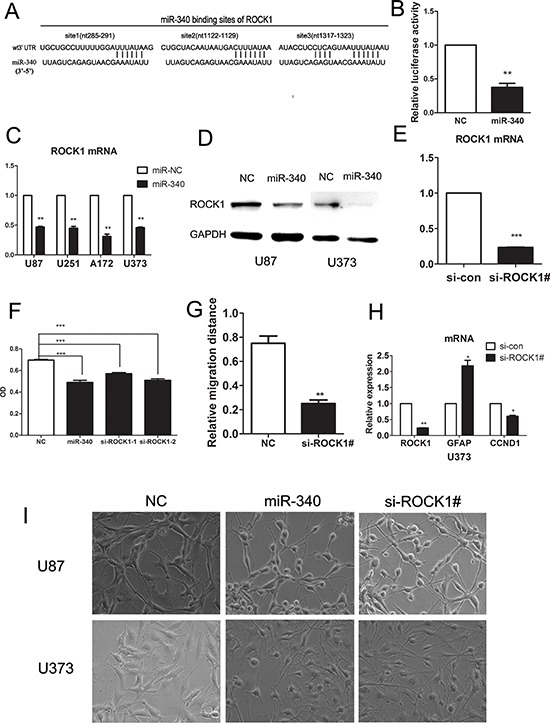
miR-340 targetes ROCK1 by binding its 3′UTR, sh-RNA-mediated knock-down of ROCK1 inhibites glioma cells growth and migration **A.** The potential interactions between miR-340 and 3 putative binding sites within the ROCK1 3′-UTR were predicted by TargetScan algorisms. Complementarities between miR-340 “seed sequence” and ROCK1 3′-UTR were displayed at the schematic diagram. **B.** Overexpression of miR-340 repressed the activity of a luciferase reporter that contained the wild-type 3′-UTR with three predicted binding sites. Cells were cotransfected with the indicated RNA duplexes and PSICHECK.2 reporters. luciferase activity was detected 48 h after transfection. The normalized luciferase activitity of NC transfectants was set as relative luciferase activity 1, so no error bar is shown for NC transfectants. **C.** Introduction of miR-340 reduced the mRNA levels of ROCK1 in several glioma cell lines. **D.** Detection of the protein levels of ROCK1 after miR-340 transfection by western blot. **E.** QRT-PCR analysis of ROCK1 expression level in U87 cells with endogenous ROCK1 expression suppressed by shRNAs. Si-ROCK1# means a mixture of two different site-targeting si-ROCK1. **F.** Sh-RNA-mediated knock down of ROCK1 inhibited the U87 cell proliferation to the extent similar to that of miR-340 transfection, conducted by MTS. **G.** Sh-RNA-mediated knock down of ROCK1 inhibited the U373 cells migratory abilitives assessed by wound healing assay. **H.** Sh-RNA-mediated knock down of ROCK1 upregulated the GFAP expression and downregulated CCND1. **I.** Sh-RNA-mediated knock down of ROCK1 promoted morphological transformation of glioma cells similar to that induced by miR-340 transfection.

Then, we examined whether ROCK1 is a direct target of miR-340. As presented in Figure 6A, there are three predictive seed sequences in the 3′-untranslated region (3′-UTR) of ROCK1. Using the psiCHECK2 luciferase reporter plasmid in which the 3′-UTR of the ROCK1 including these three predicted binding sites was cloned downstream of luciferase gene. We showed that miR-340 significantly decreased the luciferase activity of this construct to approximately 30% as compared with the control miRNA in U87 glioma cells (Figure 6B), suggesting miR-340 was directly bound to the predictive sites located in 3′-UTR of ROCK1. To obtain further evidence that ROCK1 is a miR-340 target gene in glioma, protein and mRNA of ROCK1 were examined in cells treated with miR-340. As indicated in Figure 6C and 6D, enforced expression of miR-340 in glioma cells significantly decreased expression of ROCK1 both at protein and mRNA level. Collectively, these findings strongly indicate that miR-340 is a direct target gene of miR-340 in GBM.

### MiR-340 regulates glioma cell development by downregulating ROCK1 expression

We specifically suppressed expression of ROCK1 in glioma cells using siRNA targeting ROCK1 mRNA. The silencing effect of siRNA was validated by real time PCR (Figure 6E). Knockdown of ROCK1 expression dramatically suppressed proliferation and migration of glioma cells (Figure 6F and 6G). Similar to miR-340, silencing ROCK1 induced glioma cells morphological changes toward mature process of neurocyte (Figure 6I). Furthermore, we found that knockdown of ROCK1 upregulated GFAP expression and reduced CCND1 (Figure 6H). Herein, these results suggested that inhibition of ROCK1 was the key mechanism by which miR-340 suppressed glioma development.

## DISCUSSION

Accumulated evidence demonstrates that miRNAs play essential roles in both physiological and pathological processes, including cancer initiation and development [12, 22, 27, 36]. In this study, we demonstrated that miR-340 was significantly decreased in GBM, and its expression level was conversely correlated with survival of GBM patients. Reintroduction of miR-340 remarkably alleviated malignancy of glioma through inhibition of cell proliferation, migration and invasion, induction of apoptosis and autophagy and transformation of terminal differentiation towards mature neurocytes in GBM cells. Molecular mechanism research showed that miR-340 dramatically suppressed several crucial oncogenes including p-AKT, EZH2, EGFR, XIAP and BMI1. Further investigation revealed that miR-340 suppressed glioma development by directly targeting ROCK1. Our findings highlight the significance of miR-340 deregulation in glioma development and progression and implicate miR-340 as a candidate prognosis biomarker and treatment target for GBM therapy.

MiR-340 has been involved in the progression of tumorgenesis in several types of tumors. Wu [18] et al reported that miR-340 negatively regulates c-MET expression, inhibits metastasis in breast cancer and was associated with tumor clinical stage and overall survival of breast cancer. We once tried to examine whether miR-340 has the same effect on MET in glioma cells. Interestingly, miR-340 did not influence the MET protein level in a panel of glioma cell lines (Supplementary Figure S3). We presumed that it is because miR-340 targets different genes and thus controls various cellular pathways in heterogeneous cellular environments. MiR-340 is also identified to be a tumor suppresser in colorectal cancer through inhibiting growth and metastasis by targeting PTB1 and c-MET [19, 37]. In a recent study, miR-340 was found to inhibit lung cancer cell proliferation and induce apoptosis by accumulating p27 protein through interfering the miR-221/222 interaction with p27 3′UTR, and by inducing the stabilization of p27 though targeting the key negative posttranslational regulator SKP2 [21]. Of note, miR-340 was reported previously to be epigenetically regulated in neuroblastoma and have anti-oncogenic functions on neuroblastoma cell differentiation and apoptosis. Consistent with the tumor suppressor role in these studies, miR-340 was first reported in our work to be involved in the progression of gliomagenesis, which modulated multiple malignancy of glioma including inhibiting cell growth, blocking cell cycle, reducing glioma cell motility and promoting apoptosis, autophagy and differentiation.

One miRNA manipulates many cell processes through targeting multiple genes in tissue-specific and cell-context ways. The modulation of targeted genes expression by one single miRNA may remodel the whole cell signal pathway network and impact numerous downstream genes levels. The catalytic subunit of epigenetic regulator Polycomb repressive complex 2, EZH2, functions as a transcriptional repressor for the maintenance of DNA methylation and stable repression of specific genes, including many tumor suppressors. It is found to be overexpressed and play a crucial role in tumorigenesis of many tumors including glioma. EZH2 is essential for glioblastoma cancer stem cell maintenance, blocks astroglial differentiation and promotes glioma tumorigenensis by repressing BMPR1B and BMPR1B-mediated differentiation signaling [38, 39]. We previously reported that EZH2 was upregulated in glioma and promoted glioma cells growth through inhibition of CDK4/6-pRb-E2F1 signal pathway [17]. In this report, we found that miR-340 significantly reduces EZH2 level, providing additional molecular evidence in support of the growth-suppressive and differentiation-stimulative capacity of miR-340. Moreover, downregulation of EGFR and inhibition of phosphorylation of AKT indicated that miR-340 was able to suppress the aberrant EGFR signaling which is important in glioma progression. XIAP is a powerful inhibitor of apoptosis cell death due to its direct inhibition of the activation of caspases and subsequently block of apoptosis activation [40–42]. XIAP is also reported to inhibit autophagy of human cancer cells through regulating Mdm2-p53 signalling [25]. Silencing of XIAP promotes apoptosis and reduces tumorigenecity of glioma [43]. Consistent with these researches, induction of apoptosis and autophagy of glioma cells might be attributed to downregulation of XIAP by miR-340. BMI1 acting as a polycomb group epigenetic gene silencer is highly expressed and correlated with the poor prognosis and progression of glioma patients [44]. The oncogenic BMI1 protein sustains glioma stem cell renewal, promotes cell growth and renders protection from apoptosis [26, 44, 45]. Our results found that miR-340 suppressed BMI1 protein expression in glioma cells, which is consistant with the previous report [46]. Our functional studied showed that miR-340 displayed potent anti-tumor capacity and down-regulated key oncogenic proteins in glioma. Future studies are warranted to further elucidate the mechanism by which miR-340 regulates these oncogenes expression.

ROCK1 is well-known as an essential kinase downstream of Rho GTPases in regulation of cytoskeletal rearrangement and plays important roles in cell motility [28]. Accumulated studies have suggested that aberrant RhoA-ROCK signaling pathway in tumors mainly contributes to enhance migration and invasion [30, 47]. Functional inhibition or knock-down of ROCK1 significantly promotes the terminal differentiation of chondrocyte, keratinocyte and myoblast. Of note, suppression of ROCK signaling enhances the efficacy of bone marrow-derived mesenchymal stem cells differentiation into neurons and neuroglial cells [48]. However, the possible effect of ROCK1 on differentiation in glioma has, to our knowledge, never been investigated previously. Our data showed that sh-RNA mediated knock-down of ROCK1 expression promoted morphological change and up-regulated the mature differentiation marker, indicating the new function of ROCK1 deletion inducing glioma cells toward terminal differentiation.

While it has been reported previously that miR-340 inhibits osteosarcoma cell proliferation and metastasis by directly targeting ROCK1 [20], their interaction and functional relevance have not yet been elucidated in glioma. In our study, we revealed that the reintroduction of miR-340 reduced ROCK1 expression in both mRNA and protein level. Results from luciferase reporter assays confirmed that ROCK1 represents a direct target gene of miR-340 in glioma. In addition, a significant inverse correlation between the levels of miR-340 and mRNA expression of ROCK1 was observed in glioma (Figure 6). ShRNA-mediated silencing of ROCK1 suppressed proliferation and motility, induced changes of morphology and up-regulation of GFAP, which phenocopied the effects of miR-340 on glioma. These results indicated that the anti-tumor effect of miR-340 was mediated, at least, partly through repression of ROCK1. Knock-down of ROCK1 in glioma cells did not significantly influence EZH2, EGFR, XIAP and BMI1 expression (Supplementary Figure S4), which indicates miR-340, regulates these oncogenes through distinct mechanisms bypassing ROCK1. It is not surprising due to the multifunctional targets for one miRNA in specific cellular environment. One single target could not fully elucidate the complexity and biological functions of a miRNA. Our results suggest the miR-340 regulates a complex regulatory framework to coordinately downregulate many oncogenes regulating glioma cell proliferation, migration, invasion, apoptosis and autophagy as well as terminal differentiation. The pleiotropic functions of miR-340 in glioma development require further investigation.

In summary, our study demonstrates that miR-340 controls cell growth and motility and induces the terminal differentiation through modulation of hallmarks of glioma cells, highlighting the significant role of miR-340 in tumorigenesis of glioma and suggesting potential value as a prognosis indicator and therapeutic target of glioma.

## MATERIALS AND METHODS

### Cell lines and culture

The normal human astrocytes (NHA) were purchased from ScienCell Research Laboratories (Corte Del Cedro Carlsbad, Canada) and cultured according to the manufacturer's instructions. The human U87MG, U251MG, U373, A172, U118, T98G, SHU-44 glioblastoma cell lines were obtained from China Academia Sinica cell repository, Shanghai, China. All cells were maintained cultured in DMEM (Hyclone, Logan, Utah, USA) supplemented with 10% fetal bovine serum (Hyclone) and incubated in a humidified atmosphere containing 5% CO_2_ at 37°C without antibiotics.

### GBM specimens

Fresh tumor specimens from patients with GBM and non-neoplastic brains (NNBs) tissues from patients without glioma were collected in the Department of Neurosurgery, Sun Yat-sen Memorial Hospital of Sun Yat-sen University. All the GBM tissues have been pathologically confirmed. Collected tissues for RNA detecting were immediately snap-frozen in liquid nitrogen and stored at –80°C. The study was approved by our institutes’ ethical committee and all patients gave informed consent.

### TCGA and GEO dataset with patient information

Expression of miR-340 and survival analysis of GBM patients were performed as our previous studies [49]. Briefly: Expression data of miRNA-340 and genes and the corresponding clinical information for GBM samples were downloaded from The Cancer Genome Atlas (TCGA) data portal. The level 3 data of qualified miR-340 and genes expression of 492 samples, which included 482 GBM and 10 normal samples, were directly used to assess expression differences between GBM and non-neolpastic brain (NNBs). And there were qualified clinical information of 479 GBM patients corresponding to miR-340 expression in samples. For survival analysis, patients with survival less than 30 days were excluded, since these patients might have died for reasons other than the disease itself. A total of 458 patients fitting this criterion were included for overall survival and progression free survival analysis. For stratification analysis of survival, expression values of miR-340 were sorted by ascending order. Then, quartiles of 25%, 50% and 75% of the sorted miR-340 values were set as cut-offs for low/high expression of miR-340. The survival time was expressed as mean ± SE. In addition, 78 patients with survival longer than 2 years were included for further stratification analysis of OS and PFS.

Another miRNA microarray of GEO dataset GSE25631 with 82 GBM and 5 normal brain tissue samples were also employed to assess the incidence of low levels of miR-340 within GBM samples.

### MTS assay

Cells were plated at a density of 2000 cells per well in 96-well plates overnight and transfected with 50 nM miR-340 or NC. Twenty microliters of 3-(4, 5-dimethylthiazol-2-yl)-5-(3-carboxymethoxyphenyl)-2-(4-sulfophenyl)-2H-tetrazolium inner salt (MTS, Promega, Madision, WI, USA) was added into each well containing 100 ul medium after cells were cultured for 0, 1, 2 and 3 days. The cells were then incubated at 37°C for 4 h in a humidied 5% CO2 incubator. Optical density for the cell viability was obtained at a wavelength of 490 nm using spectrophotometric analysis (BioTek, Grand Island, NY, USA).

### Cell cycle assay

20 × 10^4^ cells were seeded in 6-well plates overnight and then transfected with 50 nM miR-340 or NC. At 48 h hours post-transfection, cells were harvested, fixed with 75% ethanol and then incubated with RNase A and propidium iodide (Sigma-Aldrich, St Louis, MO, USA) for 30 min. A total of 10 000 nuclei were examined in a FACS Calibur flow cytometer (Becton-Dickinson, Franklin Lakes, New Jersey, USA), and DNA histograms were analyzed using Modifit software.

### Colony formation assay

Cells were transfected miR-340 mimics and negative control. After 48 hours of tranfection, 500 cells were plated per well of a 6-well plate and allowed to grow for 2 weeks, then fixed with methanol/acetic acid (3:1, v/v) and stained with crystal violet.

### Invasion assays

Cell invasion was determined using 24-well Matrigel invasion chambers (Becton Dickinson) in accordance with the manufacturer's instructions. Cells (5 × 10^4^) were seeded per well in the upper well of the invasion chamber in DMEM without serum. The lower chamber well contained DMEM supplemented with 10% FBS to stimulate cell invasion. After incubation for 24 h, non-invading cells were removed from the top well with a cotton swab, while the bottom cells were fixed with methanol, stained with 0.1% crystal violet, and photographed in 3 independent 10 × fields for each well.

### Would healing assay

Cell migration was analyzed using a wound-healing assay. Briefly, cells were grown to 80% confluence in 12-well plates (Corning, Cambridge, MA, USA) and then transfected with 50 nM miR-340 or NC for 2 days. An artificial wound was scratched using a standard 200 pipette tip after which the cells were further incubated. Migration into the scratched area was documented at 24 and 48 h after wounding. At vary hours, cells were photographed, and the widths of the wound lines were measured. Scratch closure was evaluated relative to the total area of wounding.

### Protein isolation and western blotting

Cells were washed twice with ice-cold phosphate-buffered saline, and lysed in RIPA buffer (Pierce, Waltham, MA, USA). Protein lysates were separated by 10–12% SDS-PAGE, and then electrophoretically transferred to PVDF membrane (Milipore, Lake Placid, NY, USA). After blocked in 5% non-fat milk or 5% BSA, the membrane was incubated with either rabbit anti-human EZH2 (1:1000, Cell Signaling Tech., Beverly, MA, USA), rabbit anti-human EGFR (1:1000, CST), rabbit anti-human AKT and p-AKT (Ser473, 1:1000, CST), mouse anti-human XIAP (1:1000, CST), rabbit anti-human p62 (1:1000, CST), rabbit anti-human LC3B (1:1000, CST), rabbit polyclonal antibody ROCK-1 (H85, 1:200 Santa Cruz, Dallas, Texas, USA), rabbit anti-human CCND1 MET, GFAP, BMI1(1:1000, abcam, Cambridge, MA, USA), rabbit anti-human GAPDH antibody (1:1000, CST) or rabbit anti-human Tubulin (1:1000, CST), followed by HRP (horseradish peroxidase)-labeled goat-anti-mouse or goat-anti-rabbit IgG (1:5000, Santa Cruz). Protein levels were detected using ECL detection solution (Pierce) and visualized on Bio-Rad ChemiDocXRS (Bio-Rad Laboratories, Hercules, CA, USA).

### Cell transfection

RNA oligos were transfected using Lipofectamine RNAiMAX (Invitrogen, Carlsbad, CA, USA), a final concentration of 50 nM duplex or 100 nM si-RNA was used. The transfection efficiency monitored by Cy3-labeled RNA mimics (RiboBio Co. Ltd, Guangzhou, China) was no less than 90% (Supplementary Figure S5). Co-transfection of the miRNA mimics and plasmid DNA was conducted using Lipofectamine 2000 (Invitrogen). All transfections were performed according to the manufacturer's protocols.

### RNA oligoribonucleotide and plasmids

All RNA oligoribonucleotides were purchased from Genepharma (Shanghai, China). The siRNAs targeting the mRNA of human ROCK1 (GenBank accession no. NM_005406) was indicated as si-ROCK1. The negative control (NC) RNA duplex for both the miR-340 mimic and the siRNA was non-homologous to any human genome sequences.

To verify the miR-340-targeted 3′-UTR, a wild-type 3′UTR segment (1615 bp) of human ROCK1 mRNA that contains three conserved putative binding site for miR-340 was cloned into the Xhol1 / BamH1 sites of 3′-UTR of the Renilla luciferase gene of the psiCHECK2 vector (Promega). The authenticity of DNA sequences was confirmed by sequencing. All RNA oligoribonucleotides and primers are listed in Supplementary Table 1.

### Luciferase reporter assay

U87 cells were seeded into 96-well plates at 10000 cells per well the day before transfection. Cells were transfected with a mixture of 100ng psiCHECK2-ROCK1–3′UTR and 50 nM miRNA mimic or negative control by using the Lipofectamine 2000 reagent according to the the manufacturer's instruction. After 48 h of transfection, firefly and Renilla luciferase activity was measured by the Dual-luciferase Reporter Assay System (Promega). The relative Renilla luciferase activities were detected by normalizing to firefly luciferase activities which served as an internal control for transfection efficiency.

### RNA extraction and quantitative real-time PCR

Total RNA was extracted using Trizol reagent (Invitrogen) according to its protocol. total RNA was reversely transcribed using PrimeScript RT Reagent Kit (Takara, Otsu, Shiga, Japan). The expression level of mature miRNAs was separately quantified with specific primers (RiboBio). Quantitative real-time PCR (qPCR) was performed using SYBR Green PCR master mix (Takara) on an Applied Biosystems StepOnePlus™ System (Applied Biosystems, Foster City, CA, USA). U6 snRNA or β-actin was used as an endogenous control. All real-time PCR reactions were performed in triplicate, and relative quantifications (RQs) were calculated using the ßßCt method (95% CI). The sequences of PCR primers were listed in Supplementary table 1.

### Apoptosis assay

Cells were transfected with indicated miRNA duplexes for 72 hours, then collected and treated with Annexin V-FITC/PI double staining (BioVision Milpitas, CA, USA). Afterward, cells were analyzed by FACS Calibur flow cytometer (Becton-Dickinson). For cell-cycle analysis, 72 hours after transfection, attached and floating cells were collected for propidium iodide (Sigma-Aldrich) staining and then tested by FACS Calibur flow cytometer (Becton-Dickinson). Sub-G1 population is expected to represent the apoptosis cell.

### Morphological evaluation

The morphologies of indicated glioma cells were monitored during a time course of 48 h and 72 h with an ECLIPSE Ti-U inverted microscope (Nikon, Tokyo, Japan) and a SIGHT DS-Qilmc CCD camera (Nikon).

### Statistical analysis

Data were imaged with GraphPad Prism 5 software (GraphPad Software, Inc, La Jolla, CA, USA). Quantitative data were expressed as mean ± s.e.m. Two group comparisons were analyzed with *t*-test and *P* values were calculated. Survival analysis was carried out with the Kaplan–Meier method. All the analyses were performed with the SPSS software (version 19.0) (IBM Corporation, New York, NY, USA). For all analyses, *P* < 0.05 was considered statistically significant.

## SUPPLEMENTARY FIGURES


