# Supplementary material for: miR-340 suppresses glioblastoma multiforme
Source: Oncotarget. 2015 Mar 16;6(11):9257–70. doi: 10.18632/oncotarget.3288 (PMC4496215; doi:10.18632/oncotarget.3288)
Supplement: Supplementary file 1 [file oncotarget-06-9257-s001.pdf]

SUPPLEMENTARY FIGURES

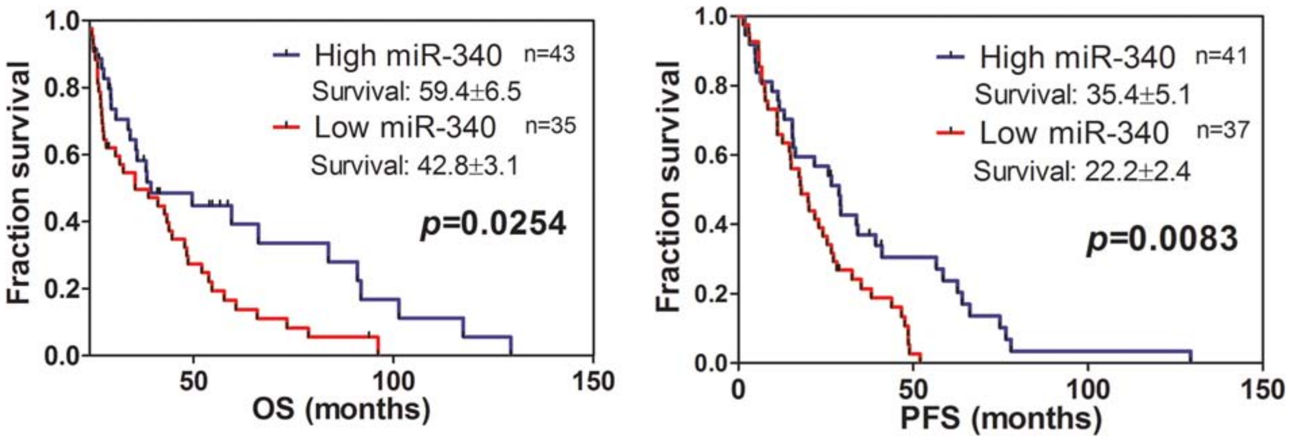

Supplementary Figure S1: OS and PFS curves of Kaplan-Meier analysis at the stratification of patients with survival longer than 2 years from TCGA data.

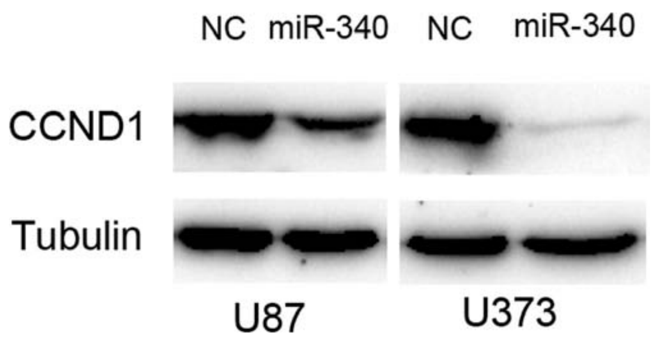

Supplementary Figure S2: MiR-340 down-regulates CCND1 expression. Westernblot analysis of CCND1 in U87 and U373 cells transfected with negative control miRNAs and miR-340 mimics.

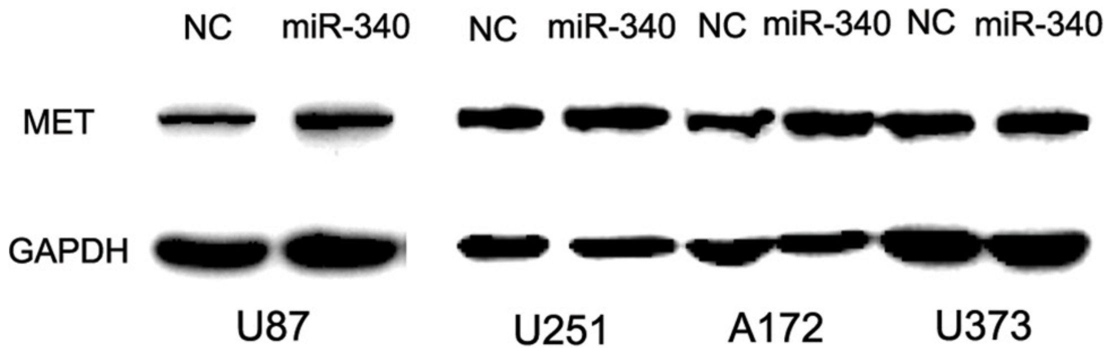

Supplementary Figure S3: MiR-340 overepression does not affect MET expression in glioma cells. Western blotting was performed on a panel of glioma cell lines tranfected with miR-340 mimic or NC.

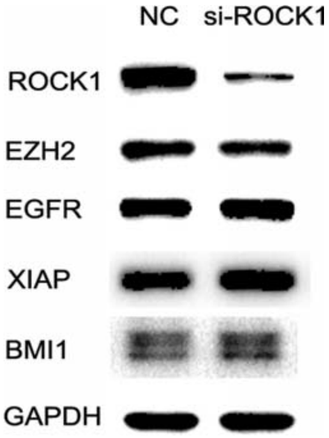

Supplementary Figure S4: Know-down of ROCK1 does not significantly affect these oncogenes expression in glioma. U87 cell were transfected with si-ROCK1 or NC, the protein expression of ROCK1, EZH2, EGFR, BMI1, XIAP were examined by Western blots.

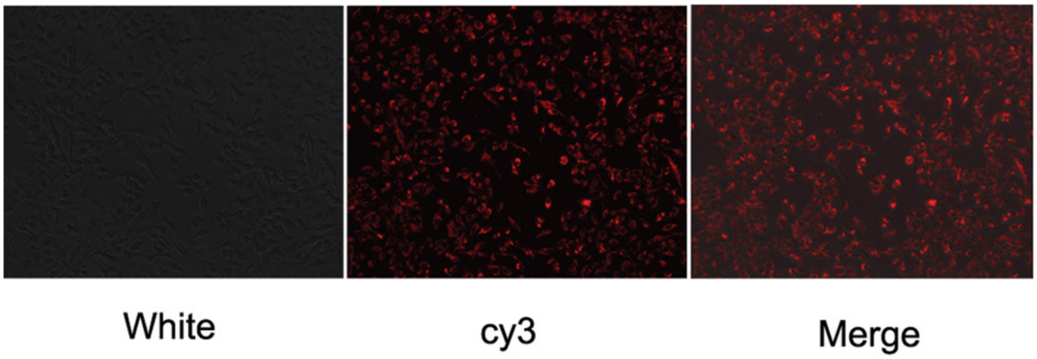

Supplementary Figure S5: The transfection efficiency of Lipofectamine RNAiMAX was monitored by Cy3-labeled RNA mimics. U251 cells were transfected with Cy3-labeled RNA mimics. Photos were captured with fluorescence microscope (Nikon) after 24 hours post transfection (Original magnification: × 200).

Supplementary Table S1. Primers, miRNA and siRNAs Sequences

**ROCK1 3′UTR cloning primers**	Forward: 5′-ATCTCGAGGAGTGCCCTGTGGAATC -3′<br>Reverse: 5′-CTGGATCCGGAATTTCACAAACATT -3′
**qPCR primers**	ROCK1 : Forward: 5′-AACCATGTGACTGAGTGCCC -3′<br>Reverse: 5′-TCAGTGTGTTGTGCCAAAGC-3′<br>beta-actin: Forward: 5′-TCATGAAGTGTGACGTGGACATC-3′<br>Reverse: 5′-CAGGAGGAGCAATGATCTTGATCT-3′<br>Dlx2: Forward: 5′-ATGTCTCCTACTCCGCCAAA -3′<br>Reverse: 5′-TGGCTTCCCGTTCACTATTC-3′<br>Brn3a: Forward: 5′-GCAGCGTGAGAAAATGAACA-3′<br>Reverse: 5′-TTTCATCCGCTTCTGCTTCT-3′<br>NeuroD6: Forward: 5′-CTGAGGATTGGCAAGAGACC-3′<br>Reverse: 5′-GCTGTGGTAGGGTGGGTAGA-3′<br>GFAP: Forward: 5′-TGCGGCTCGATCAACTCA -3′<br>Reverse: 5′-GTTGGTTTCATCCTGGAGCTTCT -3′<br>CCND1: Forward: 5′-CGTGGCCTCTAAGATGAAGG-3′<br>Reverse: 5′-CTGGCATTTTGGAGAGGAAG-3′<br>VEGF: Forward: 5′-CTACCTCCACCATGCCAAGT -3′<br>Reverse: 5′-GCAGTAGCTGCGCTGATAGA -3′<br>MMP1: Forward: 5′-CTGGCCACAACTGCCAAATG -3′<br>Reverse: 5′-CTGTCCCTGAACAGCCCAGTACTTA -3′<br>MMP2: Forward: 5′-TCTCCTGACATTGACCTTGGC -3′<br>Reverse: 5′-CAAGGTGCTGGCTGAGTAGATC -3′<br>MMP9: Forward: 5′-TTGACAGCGACAAGAAGTGG -3′<br>Reverse: 5′-GCCATTCACGTCGTCCTTAT -3′
**MiRNA and siRNA sequences**	miRNA-340 mimics: 5′-UUAUAAAGCAAUGAGACUGAUU-3′<br>siROCK1-1: 5′-AAGTAGTGACATTGATACTAG-3′<br>siROCK1-2: 5′-CAGCAAAUCCUAAUGAUAA-3′<br>Negative control :5′-UUCUCCGAACGUGUCACGU-3′
